# Supplementary material for: General anaesthesia decreases the uniqueness of brain functional connectivity across individuals and species
Source: Nat Hum Behav. 2025 Mar 24;9(5):987–1004. doi: 10.1038/s41562-025-02121-9 (PMC12106074; doi:10.1038/s41562-025-02121-9)
Supplement: Supplementary file 1 — Supplementary Figs. 1–28 and Tables 1 and 2. [file 41562_2025_2121_MOESM1_ESM.pdf]

nature human behaviour

Supplementary information https://doi.org/10.1038/s41562-025-02121-9

# General anaesthesia decreases the uniqueness of brain functional connectivity across individuals and species

In the format provided by the authors and unedited

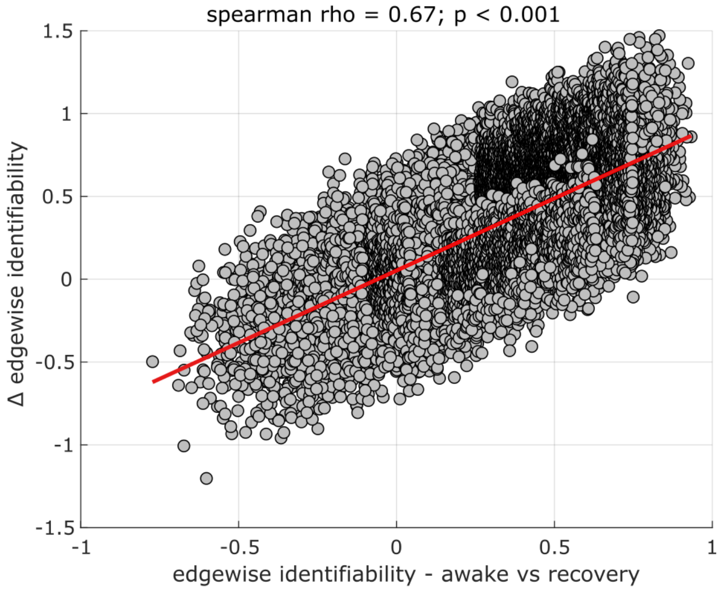


Figure S1. **Correlation between edgewise identifiability during wakefulness, and anaesthetic-induced change in edgewise identifiability |** Abscissa: edgewise identifiability (intra-class correlation) during wakefulness (awake vs recovery). Ordinate: change in edgewise identifiability under anaesthesia (3% sevoflurane). Spearman correlation: $rho = 0.67, p < 0.001$. N=19,900 pairs of cortical regions.

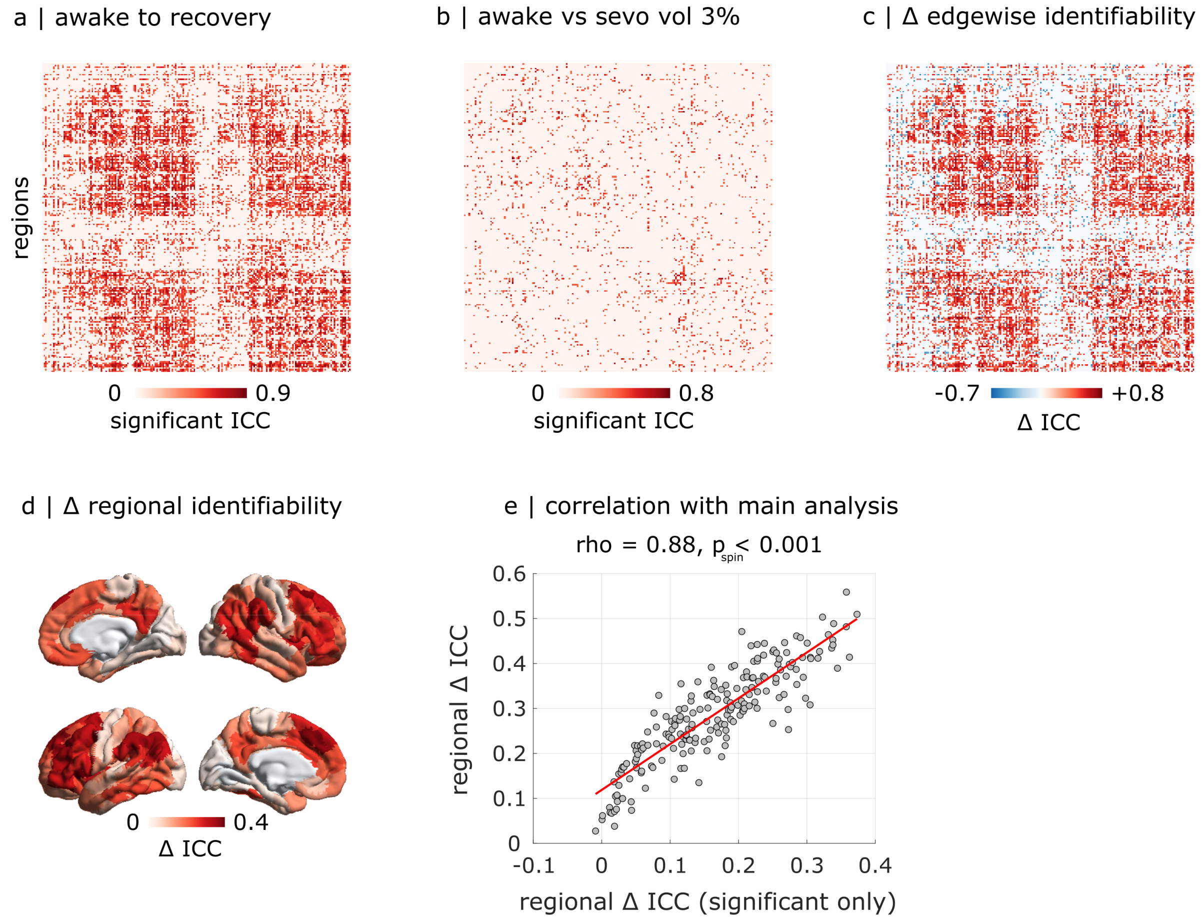


Figure S2. **Anatomical characterisation of contributions to sevoflurane-induced loss of identifiability, when only including significant ICC values|** (**a**) Significant ($p < 0.05$) edge-level intra-class correlation coefficients between awake and recovery. (**b**) Significant edge-level intra-class correlation coefficients between awake and sevoflurane $3\%$. (**c**) Difference in edge-level intra-class correlation coefficients between (a) and (b). (**d**) Regional distribution of sevoflurane-induced loss of significant ICC, projected onto the cortical surface. (**e**) The regional pattern of anaesthetic-induced changes in significant ICC is significantly spatially correlated with the corresponding map obtained when including all ICC values: Spearman correlation (two-sided): $\rho = 0.88$, $p_{spin} < 0.001$, $N = 200$ regions from the Schaefer atlas.

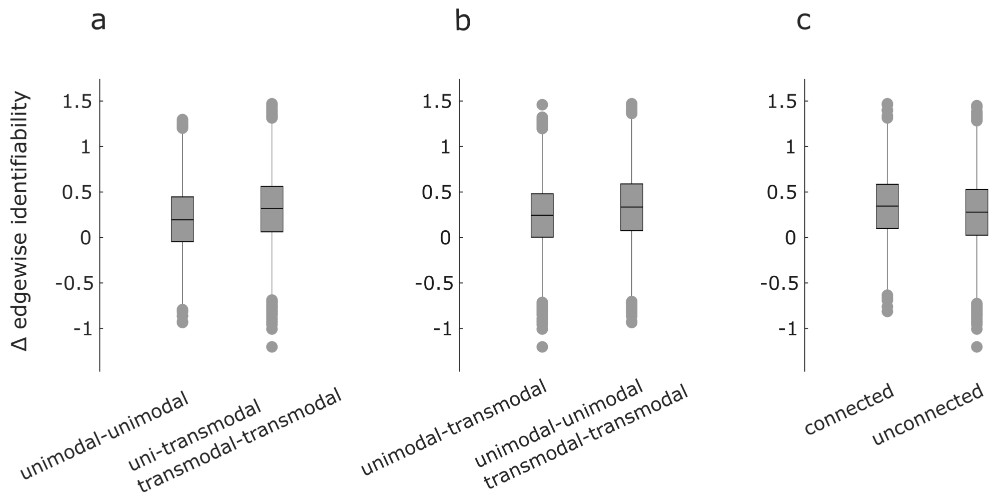


Figure S3. **Differences in edgewise identifiability changes as a function of edge type |** (**a**) Edges connecting two regions of unimodal cortex, versus other edges. Within-unimodal edges: mean $= 0.20$, SD $= 0.35$; other edges: mean $= 0.31$; SD $= 0.36$; $t(39998) = -25.09$, $p < 0.001$, effect size (Hedge's $g$) $= -0.30$, CI $[-0.32, -0.28]$. (**b**) Edges conencting unimodal and transmodal regions to each other, versus other edges (unimodal-unimodal and transmodal-transmodal). Unimodal-transmodal edges: mean $= 0.24$, SD $= 0.35$; other edges: mean $= 0.33$; SD $= 0.37$; $t(39998) = -25.43$, $p < 0.001$, effect size (Hedge's $g$) $= -0.25$, CI $[-0.27, -0.23]$. (**c**) Edges with versus without an underlying direct structural connection, as quantified using in vivo diffusion MRI tractography. Structurally connected: mean $= 0.34$, SD $= 0.35$; structurally unconnected: mean $= 0.27$; SD $= 0.36$; $t(39998) = 12.96$, $p < 0.001$, effect size (Hedge's $g$) $= 0.19$, CI $[0.16, 0.22]$. Box-plot: center line indicates the median; bounds of the box indicate the 25th and 75th percentiles; whiskers indicate $1.5\times$ interquartile range. N=40000 edges.

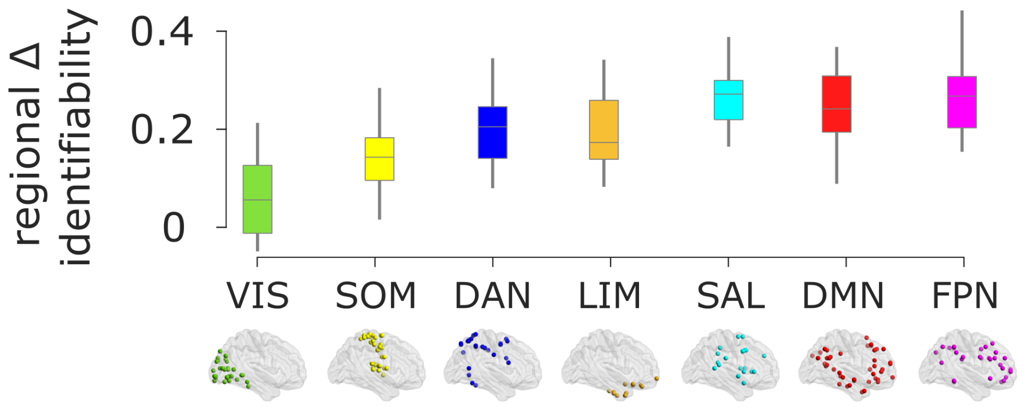


Figure S4. **Regional identifiability across intrinsic connectivity networks |**. SOM, somatomotor network; VIS, visual network; SAL, salience network; DAN, dorsal attention network; FPN, fronto-parietal control network; DMN, default mode network. Box-plot: center line indicates the median; bounds of the box indicate the 25th and 75th percentiles; whiskers indicate $1.5\times$ interquartile range. N=200 cortical regions.

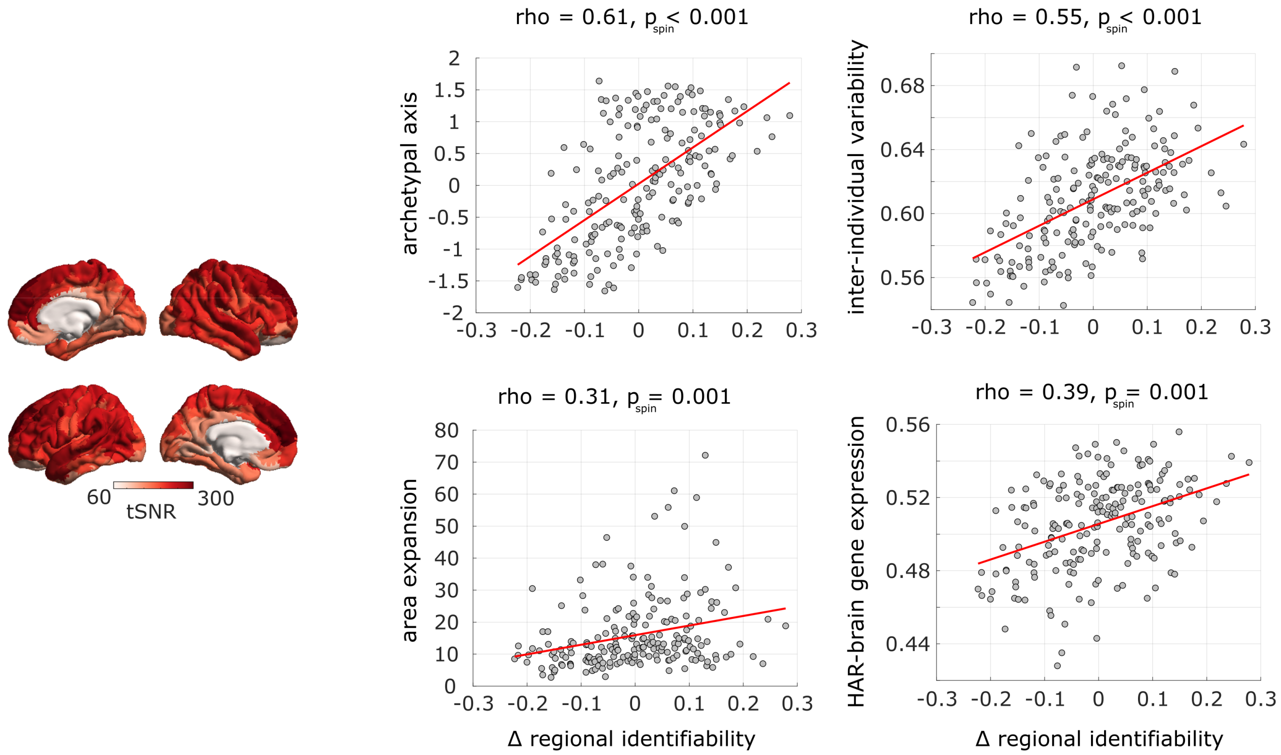


Figure S5. **Anatomical characterisation of contributions to sevoflurane-induced loss of identifiability, after regressing out regional signal-to-noise ratio of the BOLD timeseries (tSNR) | (Left)** Map of the human brain's signal-to-noise ratio of regional functional MRI signal, from [**?** ]. (**Right**) The sevoflurane-induced regional loss of ICC is significantly spatially aligned with the archetypal sensory-association axis of cortical organisation; the regional distribution of inter-individual variability of functional connectivity; the regional cortical expansion between macaque and human; and the regional expression of human-accelerated genes pertaining to brain function and development ("HAR-brain genes"), as assessed by Spearman correlation, even after regressing out the tSNR map using linear regression; $N = 200$ regions from the Schaefer atlas.

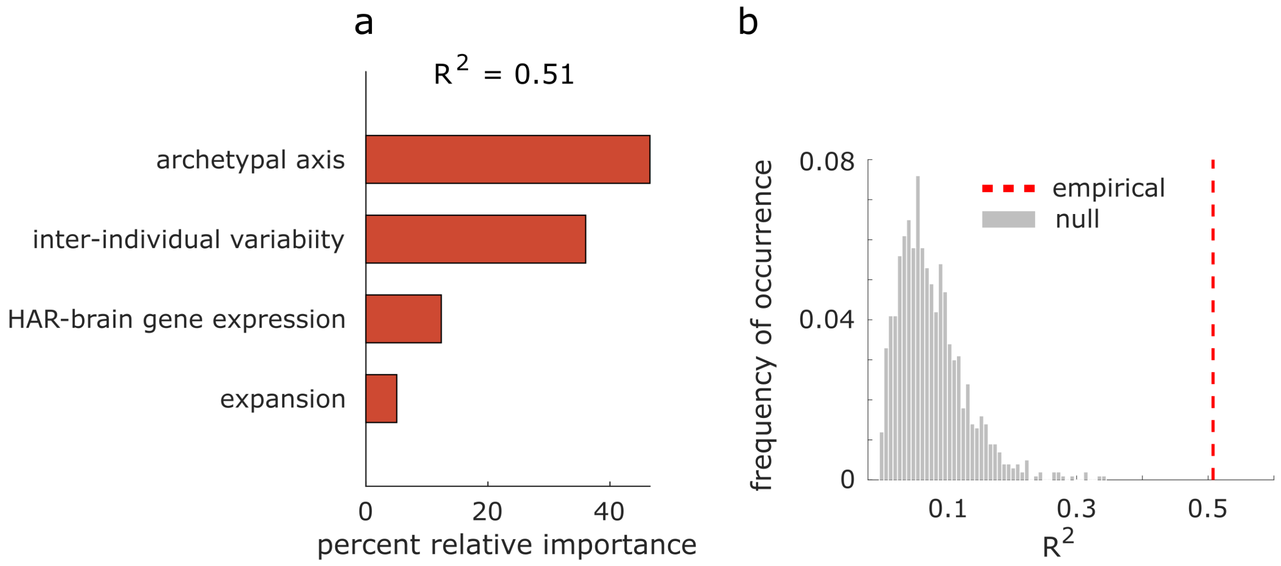


Figure S6. **Relative importance of brain maps predicting regional changes in identifiability due to $3\%$ sevoflurane anaesthesia |** (**a**) Dominance analysis compares all possible models obtained from distinct combinations of predictors, to distribute the variance explained between the predictors, in terms of percentage of relative importance (represented as bar plots). (**b**) We establish the statistical significance of our model ($p < 0.001$) using a non-parametric permutation test (one-sided), by comparing the empirical variance explained ($R^2 = 0.51$) against a null distribution of $R^2$ obtained from repeating the multiple regression with spatial autocorrelation-preserving null maps.

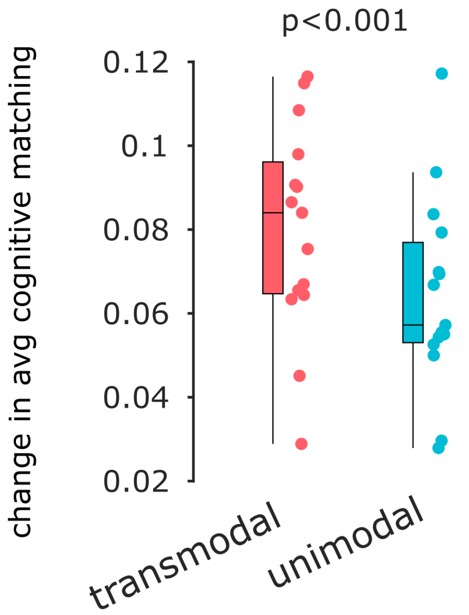


Figure S7. **Change in cognitive matching for unimodal versus transmodal ends of the cortical hierarchy|**. NeuroSynth maps were correlated with the archetypal axis map of [**?** ]; maps exhibiting a positive correlation were included at the transmodal end, whereas maps correlating negatively were included at the unimodal end. N=15 volunteers. Box-plot: center line indicates the median; bounds of the box indicate the 25th and 75th percentiles; whiskers indicate $1.5\times$ interquartile range; extreme values are shown as individual circles. ***, $p < 0.001$.

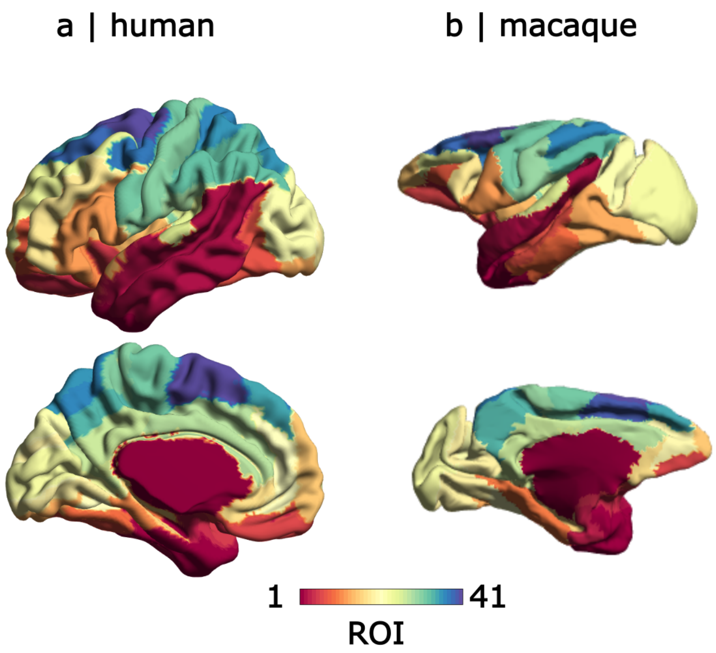


Figure S8. **Regional Mapping parcellation for different species |** (**a**) The macaque cortical atlas of Kötter and Wanke [**?** ]. (**b**) Human translation of the same atlas by [**?** ].

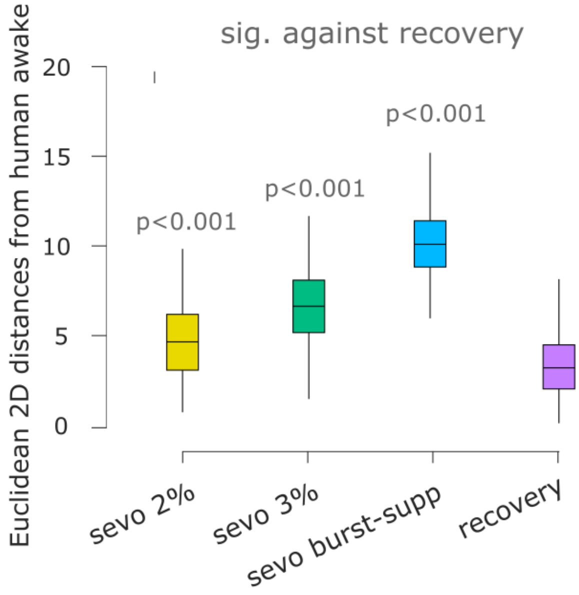


Figure S9. **Euclidean distances from human awake FC in 2D PCA space |** Distribution of Euclidean distances from awake humans' FC patterns, in the human dataset, in the space spanned by the first two principal components from Fig. 4a. N = 225 ($15 \times 15$) pairs of data-points in each box-plot. Box-plot: center line indicates the median; bounds of the box indicate the 25th and 75th percentiles; whiskers indicate $1.5\times$ interquartile range. P-values are obtained from repeated-measures t-tests (two-sided) and FDR-corrected for multiple comparisons. See Supplementary Data 2 for full statistical reporting.

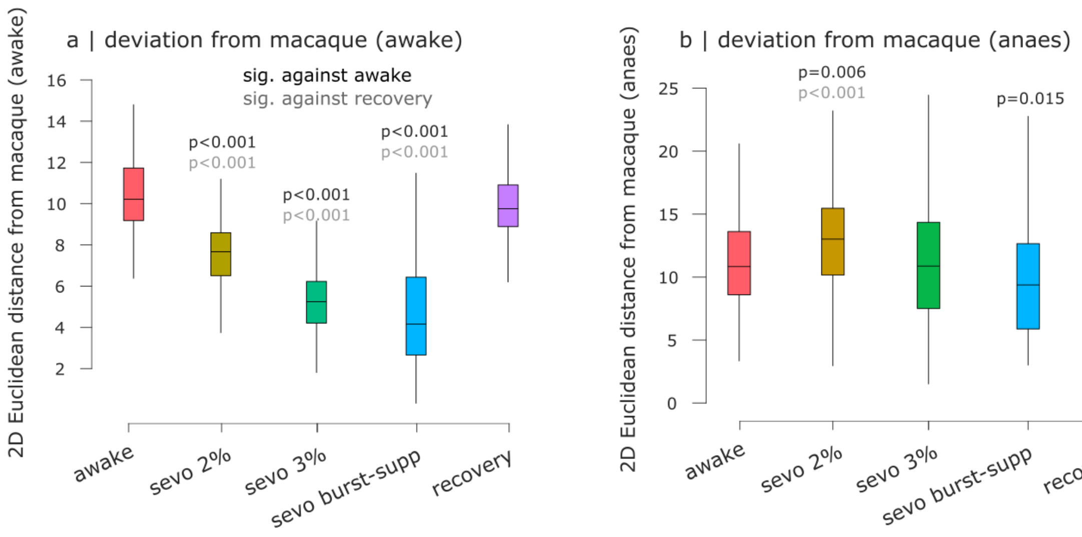


Figure S10. **Euclidean distances between human and macaque functional connectivity in 2D PCA space | (a)** Distribution of Euclidean distances from awake macaques' FC patterns, in 2D PCA space from Fig. 4a. N = 285 (15 × 19) pairs of data-points. (**b**) Distribution of Euclidean distances from anaesthetised macaques' FC patterns, in 2D PCA space. N = 135 (15 × 9) pairs of data-points. Box-plots: center line indicates the median; bounds of the box indicate the 25th and 75th percentiles; whiskers indicate 1.5× interquartile range. P-values are obtained from repeated-measures t-tests (two-sided) and FDR-corrected for multiple comparisons. See Supplementary Data 2 for full statistical reporting.

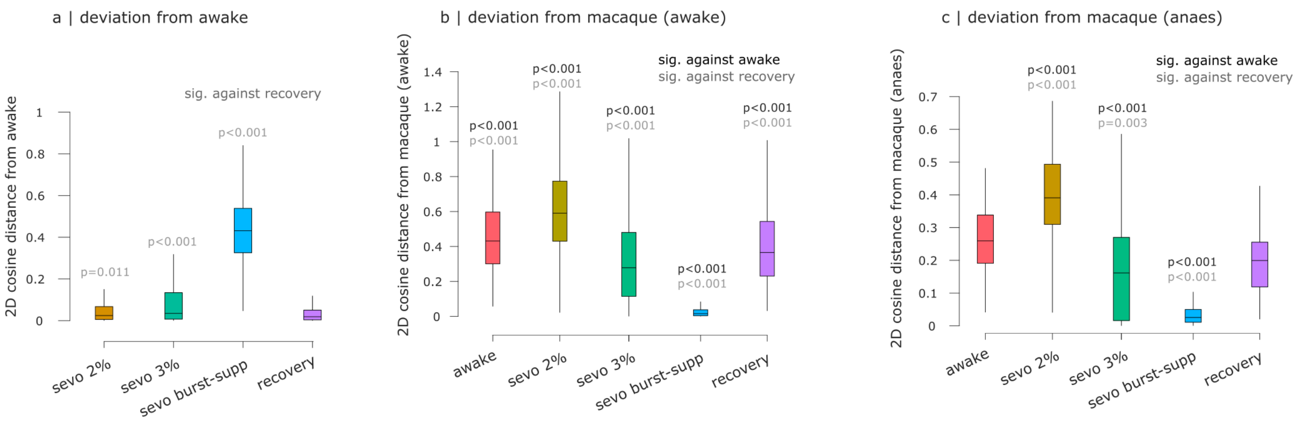


Figure S11. **Cosine distances in 2D PCA space | (a)** Distribution of cosine distances from awake humans' FC patterns, in the human dataset, in the space spanned by the first two principal components from Fig. 4a. N = 225 (15 × 15) pairs of data-points in each box-plot. (**b**) Distribution of cosine distances from awake macaques' FC patterns, in 2D PCA space. N = 285 (15 × 19) pairs of data-points. (**c**) Distribution of cosine distances from anaesthetised macaques' FC patterns, in 2D PCA space. N = 135 (15 × 9) pairs of data-points. Box-plots: center line indicates the median; bounds of the box indicate the 25th and 75th percentiles; whiskers indicate 1.5× interquartile range. P-values are obtained from repeated-measures t-tests (two-sided) and FDR-corrected for multiple comparisons. See Supplementary Data 2 for full statistical reporting.

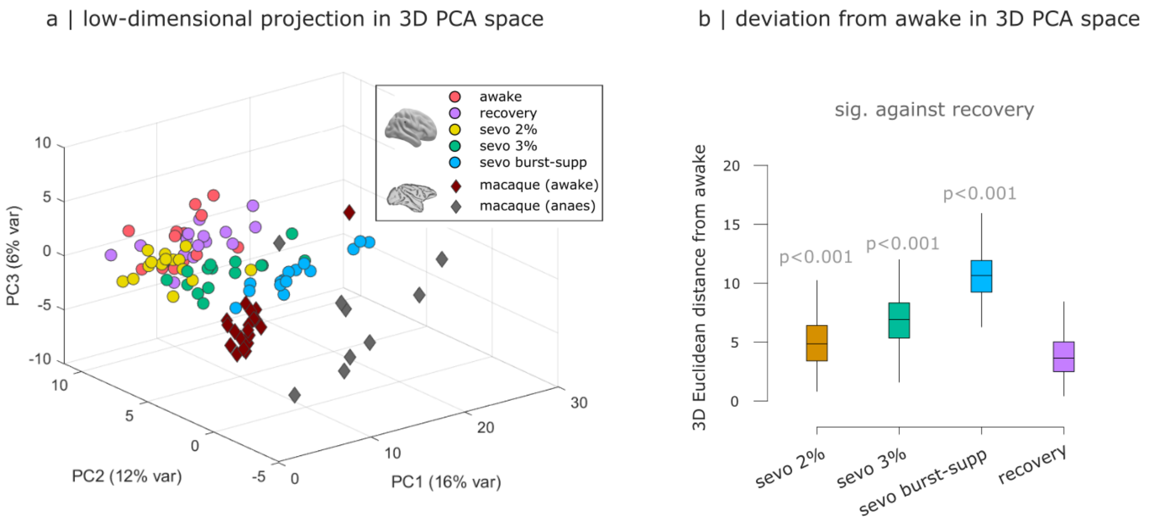


Figure S12. **Anaesthesia moves human functional connectivity closer to macaque functional connectivity in 3D PCA space | (a)** Low-dimensional projection of the human and macaque functional connectivity, in the space of the first three principal axes of variation from Principal Components Analysis. Each circle represents the FC from one human, with colour reflecting condition (awake, recovery, or different doses of sevoflurane). Each diamond represents FC from one macaque, with colour representing the dataset (awake or anaesthetised). (**b**) Distribution of Euclidean distances from awake humans' FC patterns in the human dataset, in the 3D space from (**a**). N = 225 $(15 \times 15)$ pairs of data-points. Box-plots: center line indicates the median; bounds of the box indicate the 25th and 75th percentiles; whiskers indicate $1.5\times$ interquartile range. P-values are obtained from repeated-measures t-tests (two-sided) and FDR-corrected for multiple comparisons. See Supplementary Data 2 for full statistical reporting.

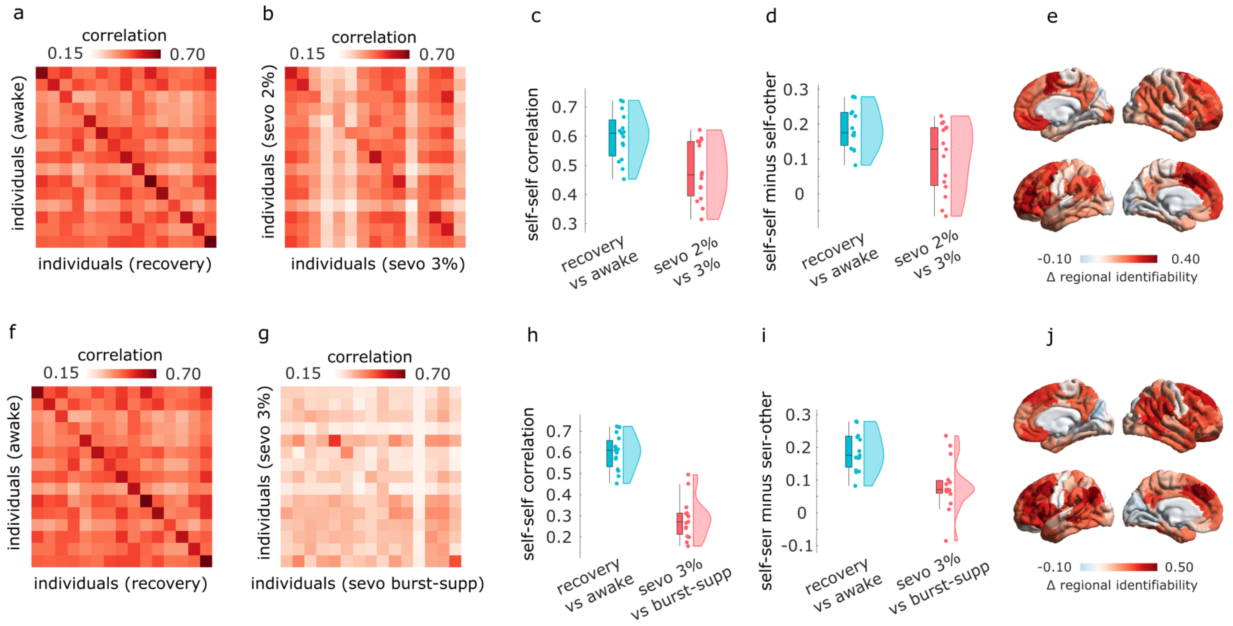


Figure S13. **Replication of identifiability results using within-state comparisons |** (**a**) Identifiability matrix between wakefulness and post-anaesthetic recovery. (**b**) Identifiability matrix between vol 2% sevoflurane and vol 3% sevoflurane anaesthesia. Entries along the diagonal, represent self-self similarity (correlation of FC patterns), whereas off-diagonal entries represent self-other similarity. (**c**) Self-self similarity is significantly higher between two conscious states, than between vol 2% and vol 3% sevoflurane. (**d**) The difference between self-self correlation and mean self-other correlation (differential identifiability) is significantly higher between two conscious states, than between vol 2% and vol 3% sevoflurane. (**e**) The regional distribution of contributions to identifiability (change in intra-class correlation coefficient) is plotted on the cortical surface. (**f**) Identifiability matrix between wakefulness and post-anaesthetic recovery. (**g**) Identifiability matrix between vol 3% and burst-suppression level of sevoflurane anaesthesia. (**h**) Self-self similarity is significantly higher between two conscious states, than between vol 3% and burst-suppression level of sevoflurane. (**i**) The difference between self-self correlation and mean self-other correlation (differential identifiability) is significantly higher between two conscious states, than between vol 3% and burst-suppression level of sevoflurane. (**j**) The regional distribution of contributions to identifiability (change in intra-class correlation coefficient) is plotted on the cortical surface. N=15 human volunteers.

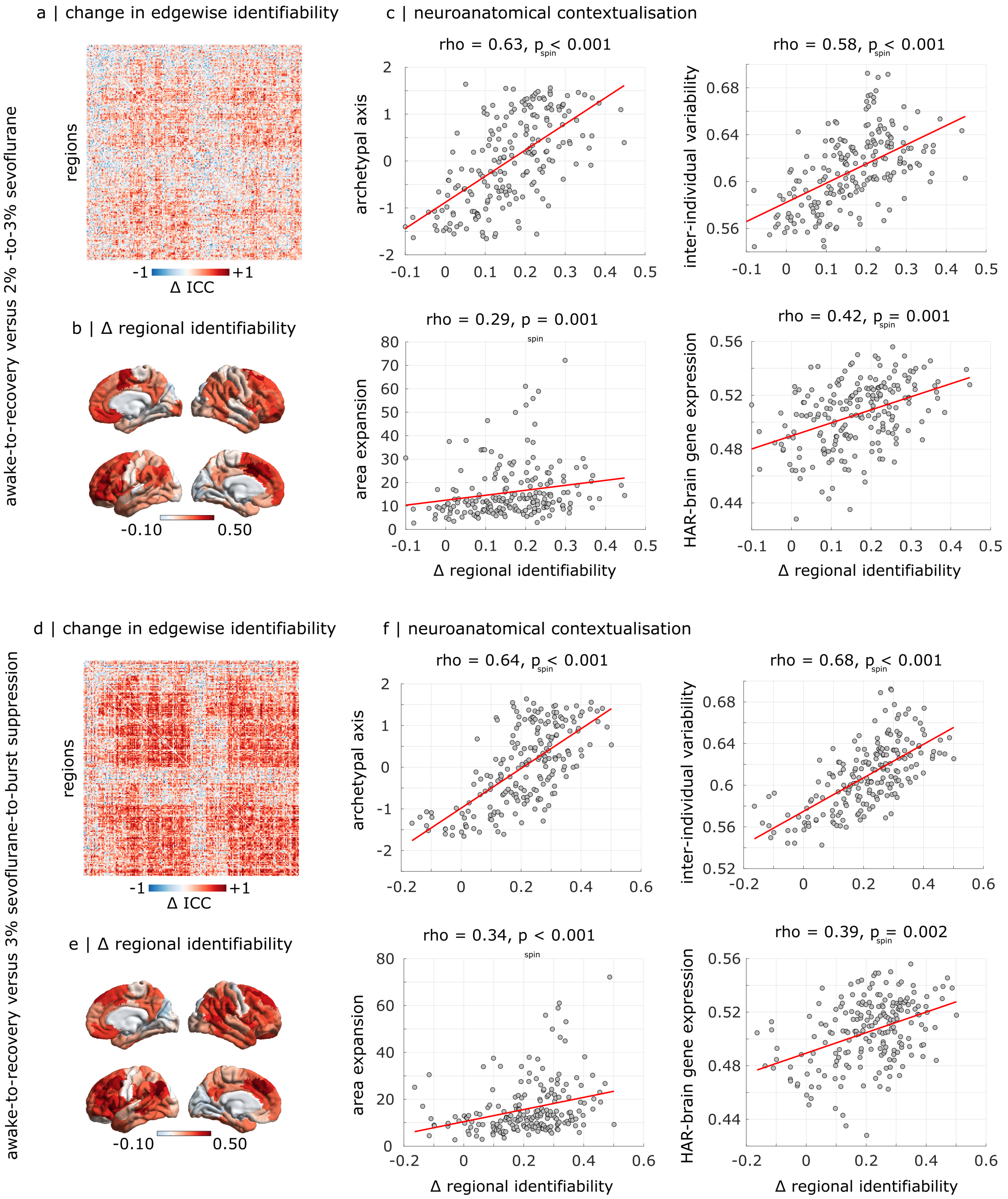


Figure S14. **Replication of edge-wise and regional identifiability results using within-state comparisons |** (**a**) Matrix of edge-level difference in intra-class correlation coefficient between awake-recovery and vol 2% - vol 3% sevoflurane anaesthesia. (**b**) Regional distribution of propofol-induced loss of ICC (awake-recovery versus 2%-3% sevoflurane). (**c**) The sevoflurane-induced regional loss of ICC (awake-recovery versus 2%-3% sevoflurane) is significantly spatially aligned with the archetypal sensory-association axis of cortical organisation; the regional distribution of inter-individual variability of functional connectivity; the regional cortical expansion between macaque and human; and the regional expression of human-accelerated genes pertaining to brain function and development ("HAR-brain genes"). (**d**) Matrix of edge-level difference in intra-class correlation coefficient between awake-recovery and vol 3% - burst-suppression levels of sevoflurane anaesthesia. (**e**) Regional distribution of propofol-induced loss of ICC (awake-recovery versus 3% sevoflurane - burst-suppression). (**f**) The sevoflurane-induced regional loss of ICC (awake-recovery versus 3% sevoflurane - burst-suppression) is significantly spatially aligned with the archetypal sensory-association axis of cortical organisation; the regional distribution of inter-individual variability of functional connectivity; the regional cortical expansion between macaque and human; and the regional expression of human-accelerated genes pertaining to brain function and development ("HAR-brain genes"). For all scatter-plots, N = 200 regions from the Schaefer atlas. P-values are obtained from Spearman correlation.

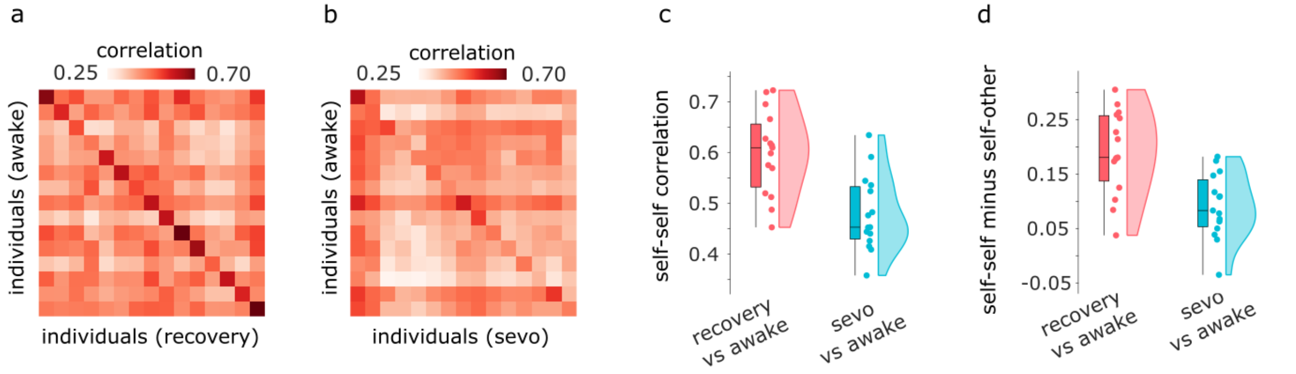


Figure S15. **Replication of identifiability results when all anaesthesia levels are concatenated |** (**a**) Identifiability matrix between wakefulness and post-anaesthetic recovery. (**b**) Identifiability matrix between wakefulness and the FC obtained after concatenating the BOLD timeseries from all three anaesthetic levels (vol 2%, vol 3%, and burst-suppression sevoflurane anaesthesia). Entries along the diagonal, represent self-self similarity (correlation of FC patterns), whereas off-diagonal entries represent self-other similarity. (**c**) Self-self similarity is significantly higher between two conscious states, than between wakefulness and the combined anaesthesia data. (**d**) The difference between self-self correlation and mean self-other correlation (differential identifiability) is significantly higher between two conscious states, than between wakefulness and the combined anaesthesia data. N=15 human volunteers.

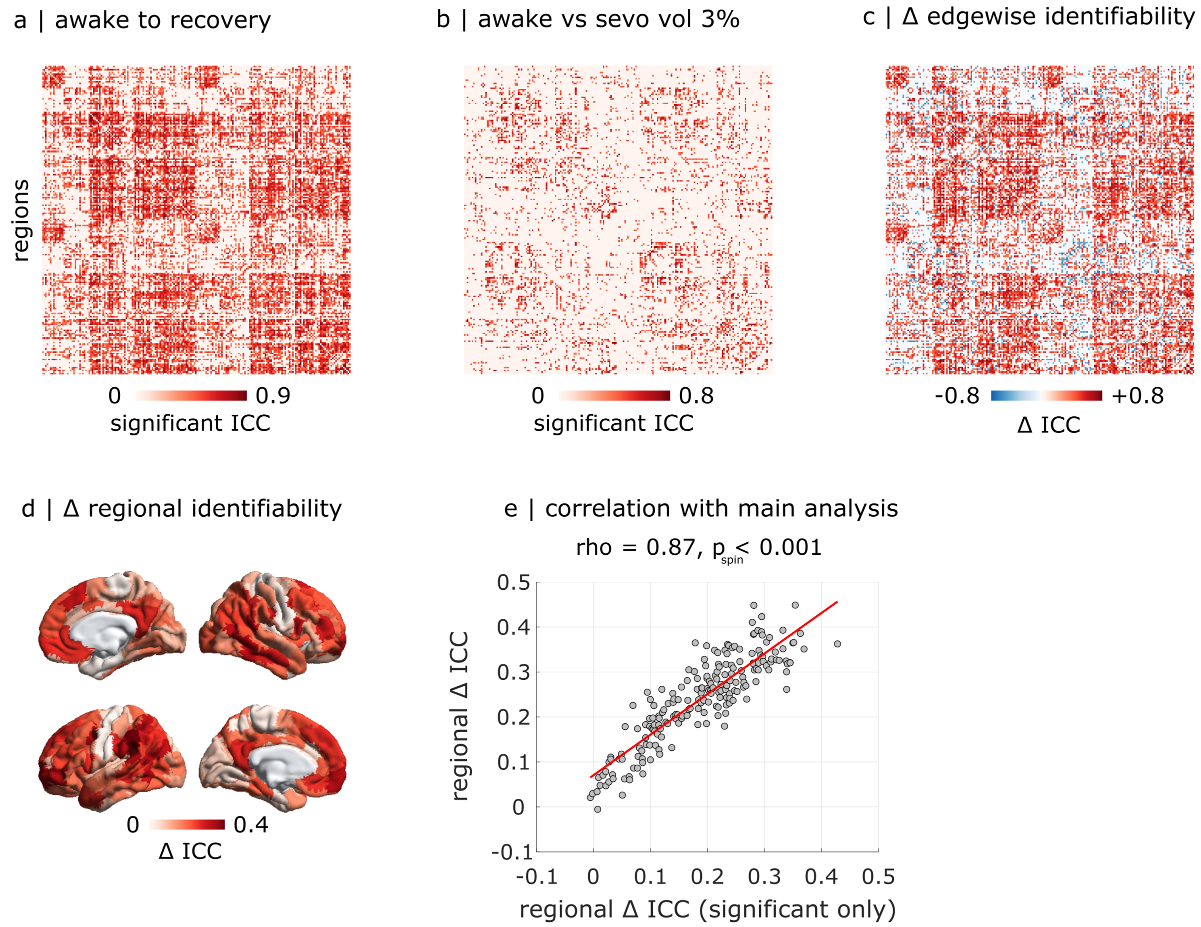


Figure S16. **Anatomical characterisation of contributions to propofol-induced loss of identifiability, when only including significant ICC values|** (**a**) Significant edge-level intra-class correlation coefficients between awake and recovery. (**b**) Significant edge-level intra-class correlation coefficients between awake and propofol. (**c**) Difference in edge-level intra-class correlation coefficients between (a) and (b). (**d**) Regional distribution of propofol-induced loss of significant ICC, projected onto the cortical surface. (**e**) The regional pattern of anaesthetic-induced changes in significant ICC is significantly spatially correlated with the corresponding map obtained when including all ICC values. Spearman correlation: $\rho = 0.87$, $p_{spin} < 0.001$, $N = 200$ regions from the Schaefer atlas.

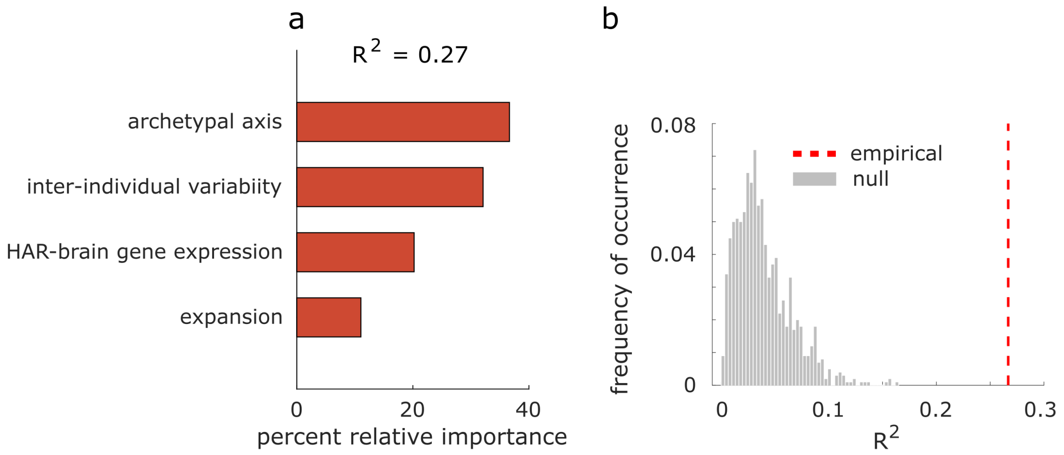


Figure S17. **Relative importance of brain maps predicting regional changes in identifiability due to propofol anaesthesia | (a)** Dominance analysis compares all possible models obtained from distinct combinations of predictors, to distribute the variance explained between the predictors, in terms of percentage of relative importance (represented as bar plots). (**b**) We establish the statistical significance of our model ($p < 0.001$) using a non-parametric permutation test (one-sided), by comparing the empirical variance explained ($R^2 = 0.27$) against a null distribution of $R^2$ obtained from repeating the multiple regression with spatial autocorrelation-preserving null maps.

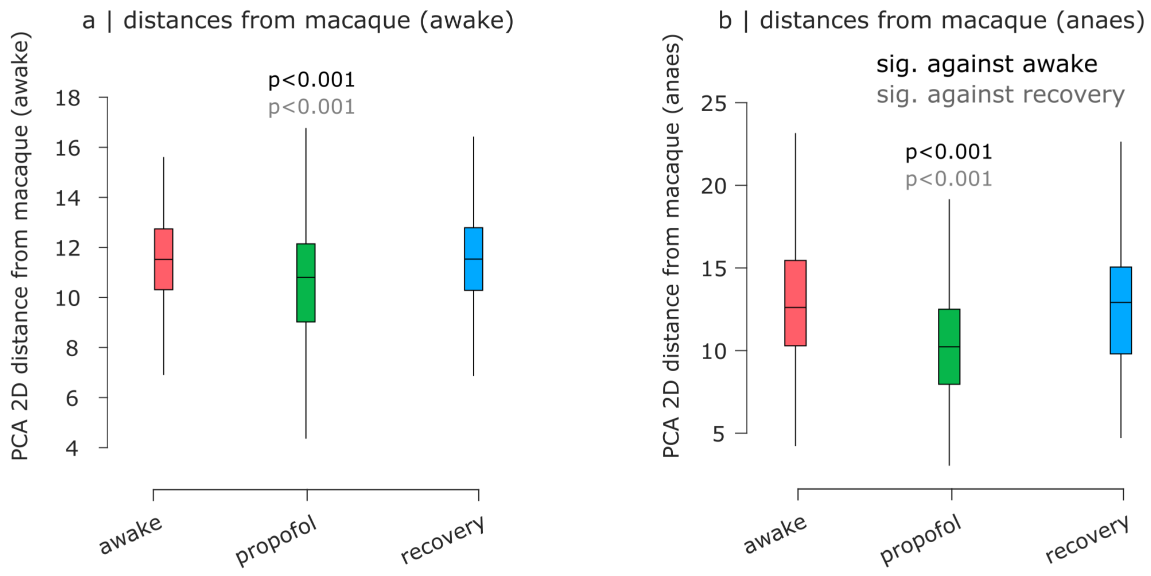


Figure S18. **Distances between human (propofol dataset) and macaque functional connectivity in 2D PCA space | (a)** Distribution of Euclidean distances from awake macaques' FC patterns, in 2D PCA space. N = 304 ($16 \times 19$) pairs of data-points. (**b**) Distribution of Euclidean distances from anaesthetised macaques' FC patterns, in 2D PCA space. N = 144 ($16 \times 9$) pairs of data-points. For c-e: box-plot center line indicates the median; bounds of the box indicate the 25th and 75th percentiles; whiskers indicate $1.5\times$ interquartile range. P-values are obtained from repeated-measures t-tests (two-sided) and FDR-corrected for multiple comparisons. See Supplementary Data 2 for full statistical reporting.

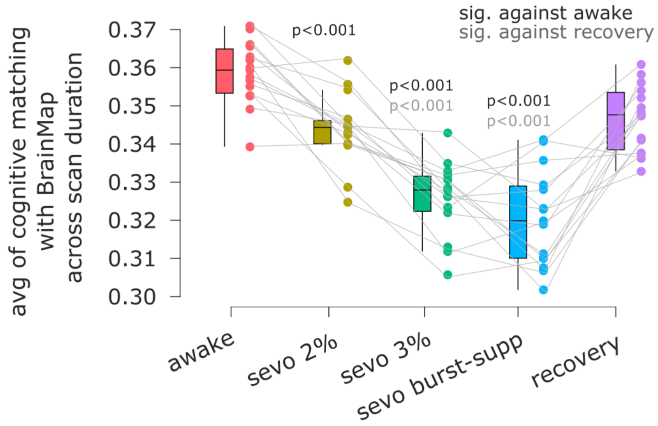


Figure S19. **Replication of cognitive matching results using the BrainMap meta-analytic database |** Ordinate: mean across time of the best decoding score (maximum spatial correlation between brain activity and 66 meta-analytic maps from BrainMap). Box-plot: center line indicates the median; bounds of the box indicate the 25th and 75th percentiles; whiskers indicate $1.5\times$ interquartile range; extreme values are shown as individual circles. N=15 human volunteers. P-values are obtained from repeated-measures t-tests (two-sided) and FDR-corrected for multiple comparisons. See Supplementary Data 1 for full statistical reporting.

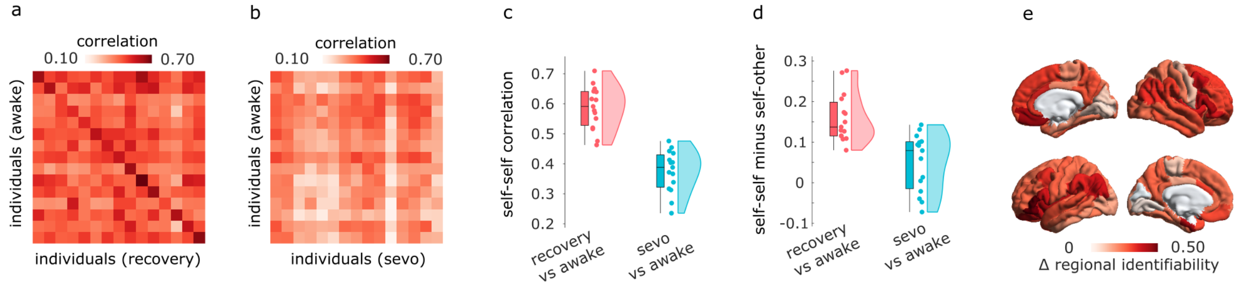


Figure S20. **Identifiability under sevoflurane anaesthesia is robust to parcellation choice |** (**a**) Identifiability matrix between wakefulness and post-anaesthetic recovery. (**b**) Identifiability matrix between wakefulness and sevoflurane anaesthesia (right). Entries along the diagonal, represent self-self similarity (correlation of FC patterns), whereas off-diagonal entries represent self-other similarity. (**c**) Self-self similarity is significantly higher between two conscious states, than between wakefulness and sevoflurane anaesthesia. (**d**) The difference between self-self correlation and mean self-other correlation (differential identifiability) is significantly higher between two conscious states, than between wakefulness and sevoflurane anaesthesia. (**e**) The regional distribution of contributions to identifiability (change in intra-class correlation coefficient) is plotted on the cortical surface for the 68 ROIs of the Desikan-Killiany atlas. Box-plot: center line indicates the median; bounds of the box indicate the 25th and 75th percentiles; whiskers indicate $1.5\times$ interquartile range; extreme values are shown as individual circles. N=15 human volunteers.

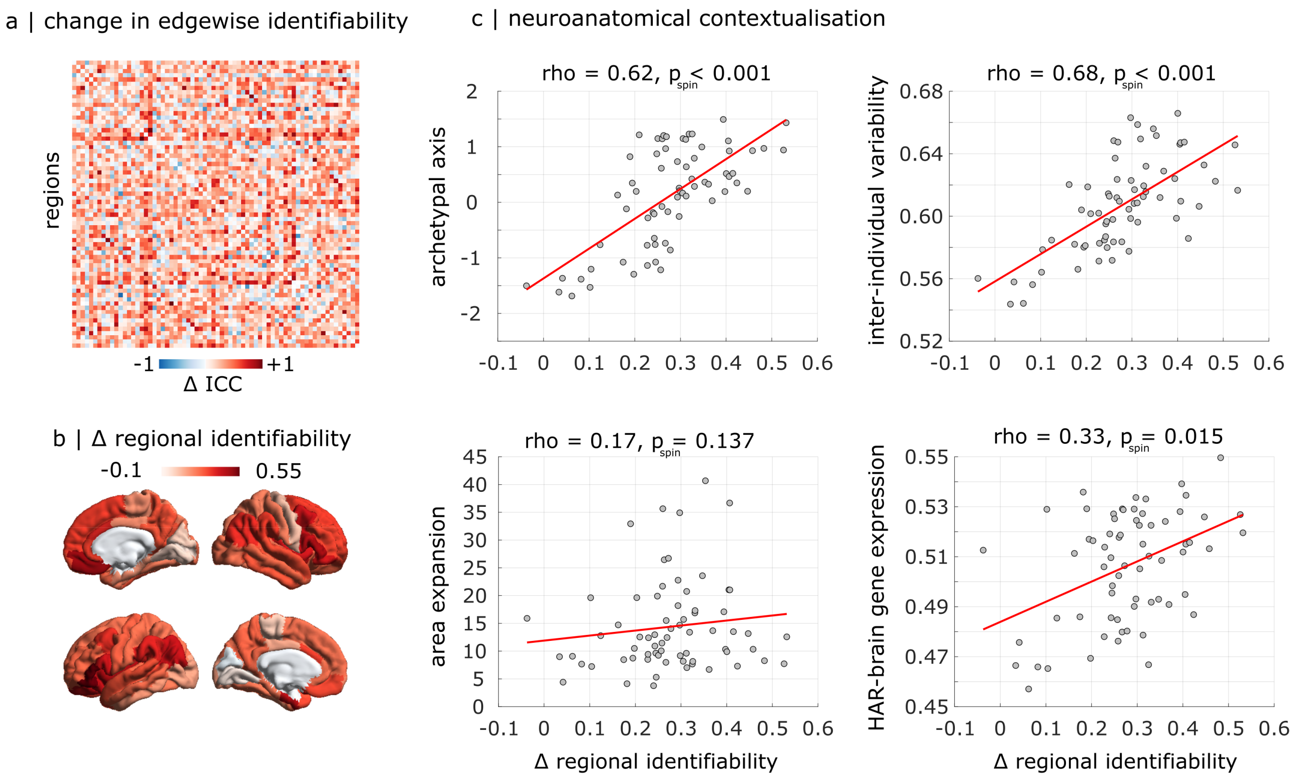


Figure S21. **Anatomical characterisation of contributions to sevoflurane-induced loss of identifiability, using the Desikan-Killiany anatomical parcellation |** (**a**) Edge-level difference in intra-class correlation coefficient between awake-recovery and awake-sevoflurane, between regions of the Desikan-Killiany anatomical atlas. (**b**) Regional distribution of sevoflurane-induced loss of ICC, projected onto the cortical surface for the Desikan-Killiany cortical anatomical atlas. (**c**) The sevoflurane-induced regional loss of ICC in Desikan-Killiany anatomical space is significantly spatially aligned with the archetypal sensory-association axis of cortical organisation; the regional distribution of inter-individual variability of functional connectivity; and the regional expression of human-accelerated genes pertaining to brain function and development ("HAR-brain genes"). P-values are obtained from Spearman correlation across $N = 68$ regions from the Desikan-Killiany atlas.

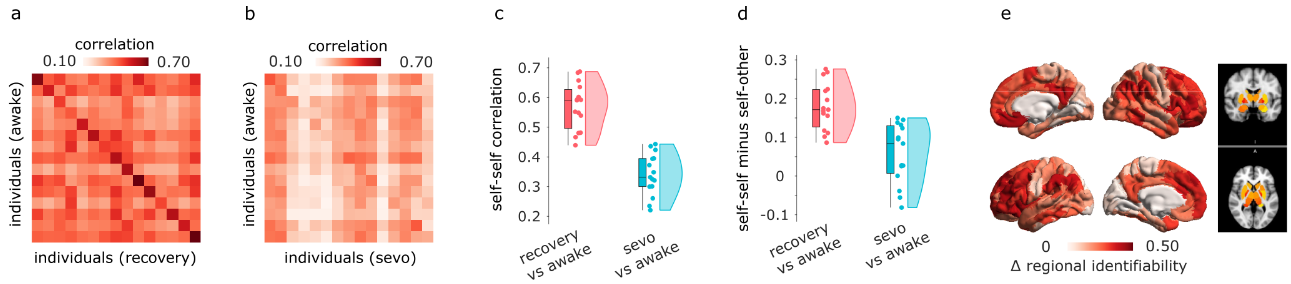


Figure S22. **Identifiability under anaesthesia is robust to inclusion of subcortex |** (**a**) Identifiability matrix between wakefulness and post-anaesthetic recovery. (**b**) Identifiability matrix between wakefulness and sevoflurane anaesthesia (right). Entries along the diagonal, represent self-self similarity (correlation of FC patterns), whereas off-diagonal entries represent self-other similarity. (**c**) Self-self similarity is significantly higher between two conscious states, than between wakefulness and sevoflurane anaesthesia. (**d**) The difference between self-self correlation and mean self-other correlation (differential identifiability) is significantly higher between two conscious states, than between wakefulness and sevoflurane anaesthesia. (**e**) The regional distribution of contributions to identifiability (change in intra-class correlation coefficient) is plotted on the cortical surface for the 200-ROIs of the Schaefer atlas, and plotted in volumetric space for the 32 ROIs of the Tian subcortical atlas. Box-plot: center line indicates the median; bounds of the box indicate the 25th and 75th percentiles; whiskers indicate $1.5\times$ interquartile range; extreme values are shown as individual circles. N=15 human volunteers.

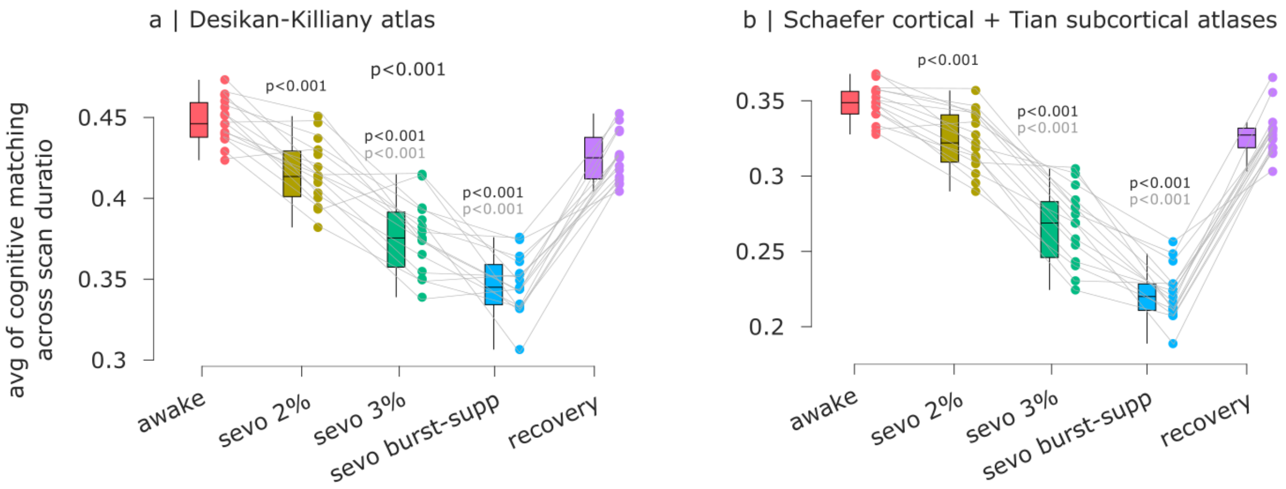


Figure S23. **Cognitive matching from meta-analytic patterns is robust to parcellation choice and inclusion of subcortex |** (**a**) Results for Desikan-Killiany anatomical parcellation. (**b**) Results for the combined Schaefer-200 cortical atlas and Tian 32-ROI subcortical atlas. Ordinate: mean across time of the best decoding score (maximum spatial correlation between brain activity and 123 NeuroSynth meta-analytic maps). Box-plots: center line indicates the median; bounds of the box indicate the 25th and 75th percentiles; whiskers indicate $1.5\times$ interquartile range; extreme values are shown as individual circles. N=15 human volunteers. P-values are obtained from repeated-measures t-tests (two-sided) and FDR-corrected for multiple comparisons. See Supplementary Data 1 for full statistical reporting.

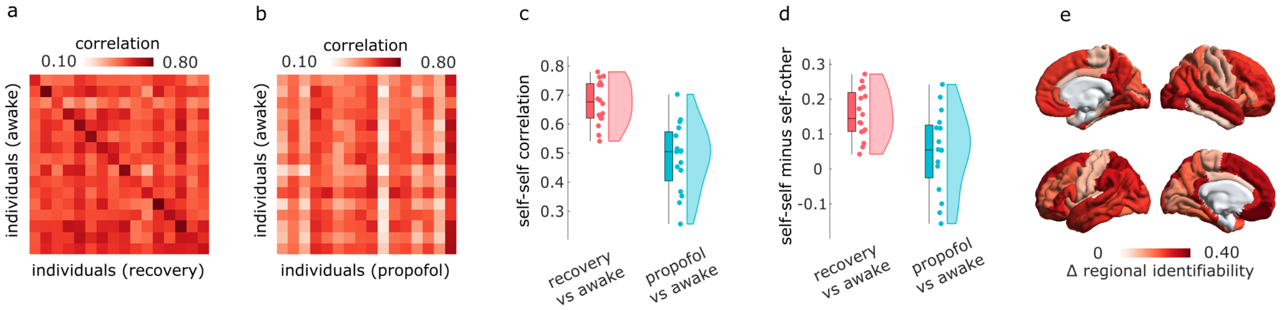


Figure S24. **Identifiability under propofol anaesthesia is robust to parcellation choice |** (**a**) Identifiability matrix between wakefulness and post-anaesthetic recovery. (**b**) Identifiability matrix between wakefulness and propofol anaesthesia (right). Entries along the diagonal, represent self-self similarity (correlation of FC patterns), whereas off-diagonal entries represent self-other similarity. (**c**) Self-self similarity is significantly higher between two conscious states, than between wakefulness and propofol anaesthesia. (**d**) The difference between self-self correlation and mean self-other correlation (differential identifiability) is significantly higher between two conscious states, than between wakefulness and propofol anaesthesia. (**e**) The regional distribution of contributions to identifiability (change in intra-class correlation coefficient) is plotted on the cortical surface for the 68 ROIs of the Desikan-Killiany atlas. Box-plot: center line indicates the median; bounds of the box indicate the 25th and 75th percentiles; whiskers indicate $1.5\times$ interquartile range; extreme values are shown as individual circles. N=16 human volunteers.

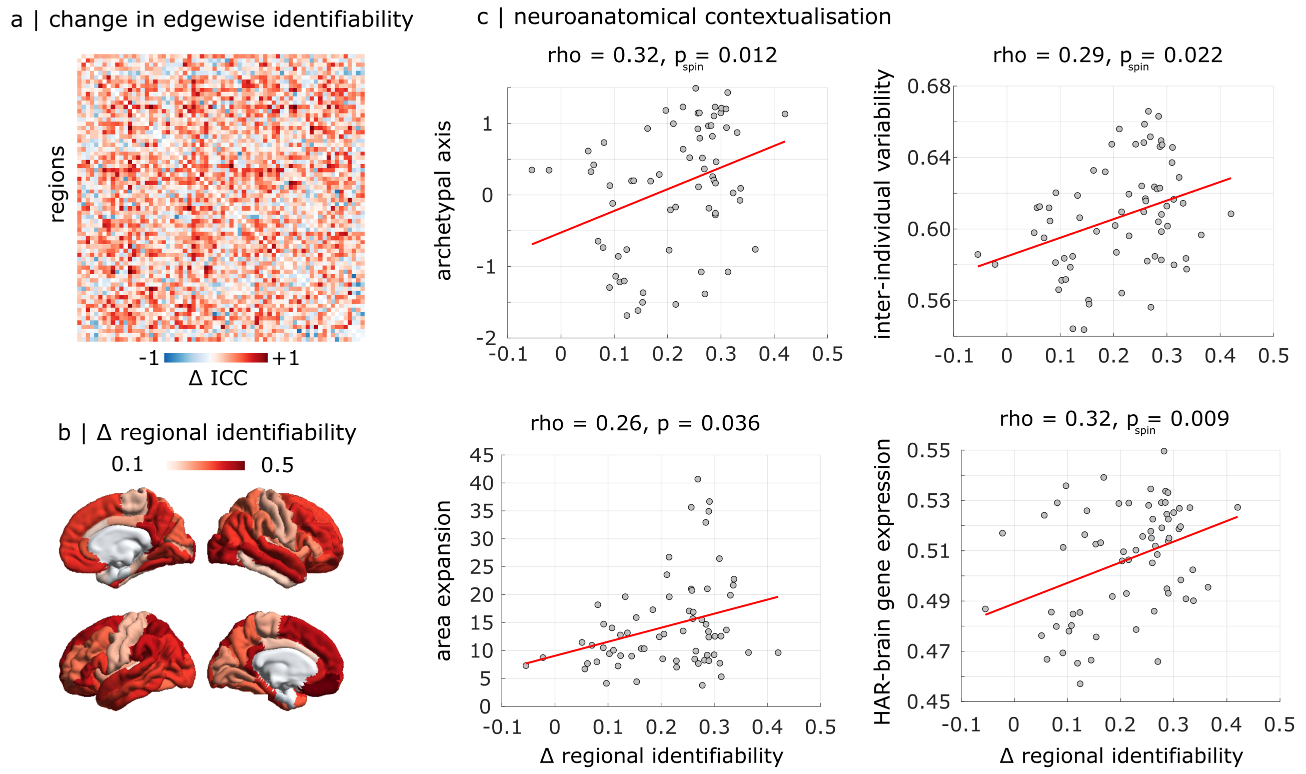


Figure S25. **Anatomical characterisation of contributions to propofol-induced loss of identifiability, using the Desikan-Killiany anatomical parcellation |** (**a**) Edge-level difference in intra-class correlation coefficient between awake-recovery and awake-propofol, between regions of the Desikan-Killiany anatomical atlas. (**b**) Regional distribution of propofol-induced loss of ICC, projected onto the cortical surface for the Desikan-Killiany cortical anatomical atlas. (**c**) The propofol-induced regional loss of ICC in Desikan-Killiany anatomical space is significantly spatially aligned with the archetypal sensory-association axis of cortical organisation; the regional distribution of inter-individual variability of functional connectivity; the regional cortical expansion between macaque and human; and the regional expression of human-accelerated genes pertaining to brain function and development ("HAR-brain genes"). P-values are obtained from Spearman correlation across $N = 68$ regions from the Desikan-Killiany atlas.

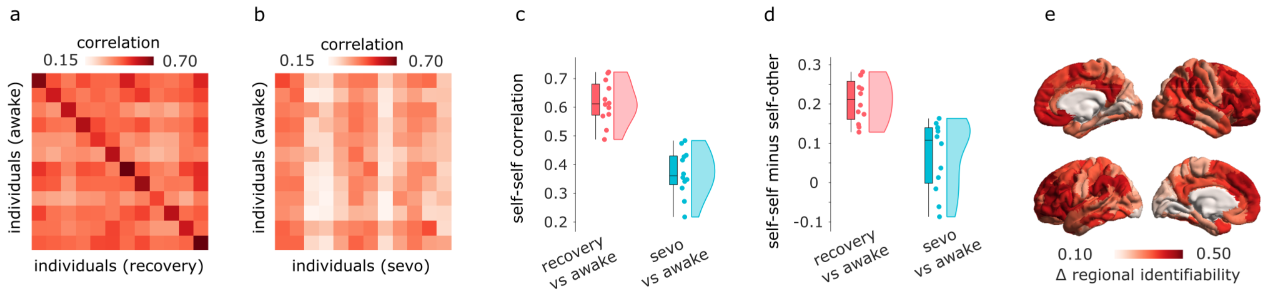


Figure S26. **Replication of identifiability results upon excluding high-motion individuals |** (**a**) Identifiability matrix between wakefulness and post-anaesthetic recovery. (**b**) Identifiability matrix between wakefulness and sevoflurane anaesthesia. Entries along the diagonal, represent self-self similarity (correlation of FC patterns), whereas off-diagonal entries represent self-other similarity. (**c**) Self-self similarity is significantly higher between two conscious states, than between wakefulness and sevoflurane. (**d**) The difference between self-self correlation and mean self-other correlation (differential identifiability) is significantly higher between two conscious states, than between wakefulness and sevoflurane. (**e**) The regional distribution of contributions to identifiability (change in intra-class correlation coefficient) is plotted on the cortical surface. It is significantly spatially correlated with the corresponding map obtained when including all individuals: Spearman $\rho = 0.94$, $p_{spin} < 0.001$, $N = 200$ regions. N=12 human volunteers.

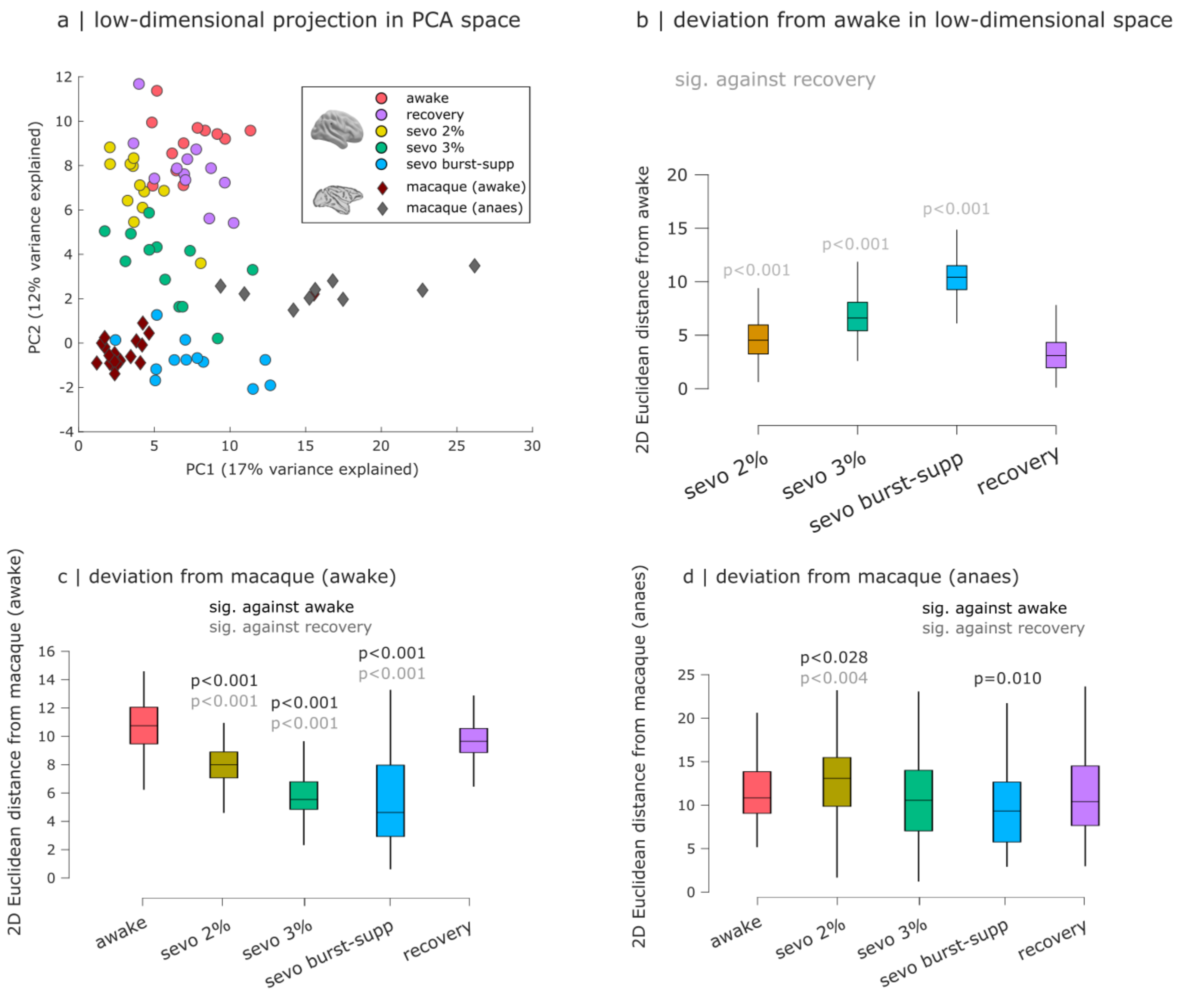


Figure S27. **Replication of PCA results upon excluding high-motion individuals | (a)** Low-dimensional projection of the human and macaque functional connectivity, in the space of the first two principal axes of variation from Principal Components Analysis. Each circle represents the FC from one human, with colour reflecting condition (awake, recovery, or different doses of sevoflurane). Each diamond represents FC from one macaque, with colour representing the dataset (awake or anaesthetised). (**b**) Distribution of Euclidean distances from awake humans' FC patterns, in the 2D space from (**a**). N = 144 (12 × 12) pairs of data-points for human data. (**c**) Distribution of Euclidean distances between the human data and awake macaques' FC patterns. N = 228 (12 × 19) pairs of data-points. (**d**) Distribution of Euclidean distances between the human data and anaesthetised macaques' FC patterns. N = 108 (12 × 9) pairs of data-points. Box-plots: center line indicates the median; bounds of the box indicate the 25th and 75th percentiles; whiskers indicate 1.5× interquartile range. P-values are obtained from repeated-measures t-tests (two-sided) and FDR-corrected for multiple comparisons. See Supplementary Data 2 for full statistical reporting.

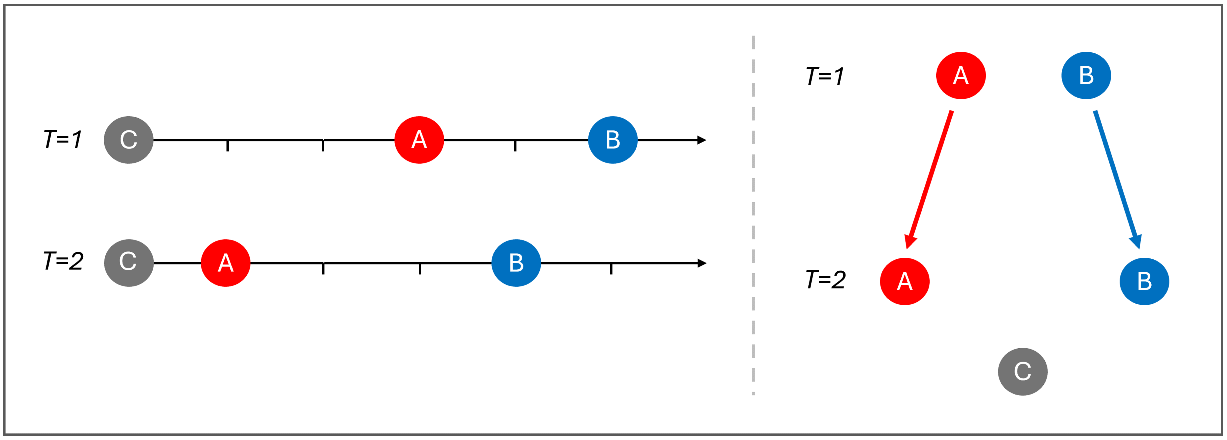


Figure S28. **Simple visual examples of how two objects can become more similar to a third, while also becoming less similar to each other |** For both the 1D example (left) and 2D example (right), proximity reflects similarity. At T=2, both A and B are closer to C than they were at T=1; however, the distance between them has increased. This phenomenon is not constrained to low-dimensional scenarios: Supplementary Code 1 provides MATLAB code for a toy example of two matrices that decrease their correlation with each other, while each increasing their correlation with a third matrix C.

| | | | | |
|---|---|---|---|---|
| action | adaptation | addiction | anticipation | anxiety |
| arousal | association | attention | autobiographical memory | balance |
| belief | categorization | cognitive control | communication | competition |
| concept | consciousness | consolidation | context | coordination |
| decision | decision making | detection | discrimination | distraction |
| eating | efficiency | effort | emotion | emotion regulation |
| empathy | encoding | episodic memory | expectancy | expertise |
| extinction | face recognition | facial expression | familiarity | fear |
| fixation | focus | gaze | goal | hyperactivity |
| imagery | impulsivity | induction | inference | inhibition |
| insight | integration | intelligence | intention | interference |
| judgment | knowledge | language | language comprehension | learning |
| listening | localization | loss | maintenance | manipulation |
| meaning | memory | memory retrieval | mental imagery | monitoring |
| mood | morphology | motor control | movement | multisensory |
| naming | navigation | object recognition | pain | perception |
| planning | priming | psychosis | reading | reasoning |
| recall | recognition | rehearsal | reinforcement learning | response inhibition |
| response selection | retention | retrieval | reward anticipation | rhythm |
| risk | rule | salience | search | selective attention |
| semantic memory | sentence comprehension | skill | sleep | social cognition |
| spatial attention | speech perception | speech production | strategy | strength |
| stress | sustained attention | task difficulty | thought | uncertainty |
| updating | utility | valence | verbal fluency | visual attention |
| visual perception | word recognition | working memory | | |

TABLE S1. **NeuroSynth terms. |** Terms that overlapped between the NeuroSynth database [**?** ] and the Cognitive Atlas [**?** ] were included in the analysis.

| | | | | |
|---|---|---|---|---|
| air-hunger | disgust | language | phonology | speech (action) |
| alcohol | emotion | learning | preparation | speech (language) |
| amphetamines | estrogen | marijuana | psychiatric medications | SSRIs |
| anger | execution | memory | reasoning | steroids and hormones |
| antidepressants | explicit | motion | rest | syntax |
| antipsychotics | fear | music | sadness | thermoregulation |
| anxiety | gustation | nicotine | semantics | thirst |
| attention | happiness | non-steroidal anti-inflammatory drugs | sexuality | time |
| audition | humour | observation | shape | vision |
| bladder | hunger | olfaction | sleep | working memory |
| caffeine | imagination | opioids | social cognition | |
| capsaicin | inhibition | orthography | soma | |
| cognition | interoception | pain | somesthesis | |
| colour | ketamine | pharmacology | space | |

TABLE S2. **BrainMap terms |** BainMap terms are organized by behavioural domain. All 66 unique behavioural domain (excluding any undefined domains) used in analyses are shown here.
