# Supplementary material for: General anaesthesia decreases the uniqueness of brain functional connectivity across individuals and species
Source: Nat Hum Behav. 2025 Mar 24;9(5):987–1004. doi: 10.1038/s41562-025-02121-9 (PMC12106074; doi:10.1038/s41562-025-02121-9)
Supplement: Supplementary file 2 — Reporting Summary [file 41562_2025_2121_MOESM2_ESM.pdf]

nature portfolio

Corresponding author(s): Andrea Luppi

Last updated by author(s): Jan 1, 2025

Reporting Summary

Nature Portfolio wishes to improve the reproducibility of the work that we publish. This form provides structure for consistency and transparency in reporting. For further information on Nature Portfolio policies, see our Editorial Policies and the Editorial Policy Checklist.

Statistics

For all statistical analyses, confirm that the following items are present in the figure legend, table legend, main text, or Methods section.

n/a	Confirmed	
☐	☒	The exact sample size (*n*) for each experimental group/condition, given as a discrete number and unit of measurement
☐	☒	A statement on whether measurements were taken from distinct samples or whether the same sample was measured repeatedly
☐	☒	The statistical test(s) used AND whether they are one- or two-sided<br>*Only common tests should be described solely by name; describe more complex techniques in the Methods section.*
☐	☒	A description of all covariates tested
☐	☒	A description of any assumptions or corrections, such as tests of normality and adjustment for multiple comparisons
☐	☒	A full description of the statistical parameters including central tendency (e.g. means) or other basic estimates (e.g. regression coefficient) AND variation (e.g. standard deviation) or associated estimates of uncertainty (e.g. confidence intervals)
☐	☒	For null hypothesis testing, the test statistic (e.g. *F*, *t*, *r*) with confidence intervals, effect sizes, degrees of freedom and *P* value noted<br>*Give P values as exact values whenever suitable.*
☒	☐	For Bayesian analysis, information on the choice of priors and Markov chain Monte Carlo settings
☒	☐	For hierarchical and complex designs, identification of the appropriate level for tests and full reporting of outcomes
☐	☒	Estimates of effect sizes (e.g. Cohen's *d*, Pearson's *r*), indicating how they were calculated

Our web collection on statistics for biologists contains articles on many of the points above.

Software and code

Policy information about availability of computer code

Data collection	The TIVA Trainer (European Society for Intravenous Aneaesthesia, eurosiva.eu) pharmacokinetic simulation program was used to control the propofol infusion. An Alaris PK infusion pump (Carefusion, Basingstoke, UK) was used for propofol infusion.
Data analysis	We have made code for cognitive matching freely available at https://github.com/netneurolab/luppi-cognitive-matching<br>The following third-party code was used.<br>Code for brain fingerprinting is freely available at https://github.com/eamico/MEG_fingerprints.<br>The code for spin-based permutation testing of cortical correlations is freely available at https://github.com/frantisekvasa/rotate_parcellation.<br>DSI Studio is freely available at https://dsi-studio.labsolver.org/.<br>The CONN toolbox is freely available at http://www.nitrc.org/projects/conn.<br>The Pypreclin code is available at https://github.com/neurospin/pypreclin.<br>The abagen toolbox is available at https://abagen.readthedocs.io/<br>The neuromaps toolbox is available at https://netneurolab.github.io/neuromaps/.<br>The Measures of Effect Size Toolbox for MATLAB is freely available at https://github.com/hhentschke/measures-of-effect-size-toolbox.<br>Supplementary Code 1 and Supplementary Code 2 are provided as supplementary materials.

For manuscripts utilizing custom algorithms or software that are central to the research but not yet described in published literature, software must be made available to editors and reviewers. We strongly encourage code deposition in a community repository (e.g. GitHub). See the Nature Portfolio guidelines for submitting code & software for further information.

Data

Policy information about availability of data

All manuscripts must include a data availability statement. This statement should provide the following information, where applicable:

- Accession codes, unique identifiers, or web links for publicly available datasets
- A description of any restrictions on data availability
- For clinical datasets or third party data, please ensure that the statement adheres to our policy

Source data are provided as a source data file.
The original pharmacological fMRI data are available from the corresponding authors of the original publications referenced herein. The Allen Human Brain Atlas transcriptomic database is available at https://human.brain-map.org/; NeuroSynth is available at https://neurosynth.org/. The list of human-accelerated brain genes is available from the Supplementary Material of [93]. The Newcastle macaque fMRI data are available from the PRIMEDE database (http://fcon_1000.projects.nitrc.org/indi/indiPRIME.html). Diffusion MRI data for the Human Connectome Project in DSI Studio-compatible format are available at http://brain.labsolver.org/diffusion-mri-templates/hcp-842-hcp-1021. Macaque fMRI data from TheVirtualBrain project [42] are available at https://openneuro.org/datasets/ds001875/versions/1.0.3.

Research involving human participants, their data, or biological material

Policy information about studies with human participants or human data. See also policy information about sex, gender (identity/presentation), and sexual orientation and race, ethnicity and racism.

Reporting on sex and gender	Each dataset had been previously collected. One dataset only included male participants; the other included participants of both sexes. The design is within-subjects, and our focus was not on comparing groups or inter-individual differences but rather on comparing states of anaesthesia.
Reporting on race, ethnicity, or other socially relevant groupings	No grouping by race, ethnicity, or socieconomic status was performed.
Population characteristics	See Life sciences reporting.
Recruitment	Sevoflurane dataset: Data acquisition took place between June and December 2013. Participants approaches the research team to seek participation. healthy adult men were recruited through campus notices and personal contact, and compensated for their participation in the study. Further exclusion criteria were the following: physical status other than American Society of Anesthesiologists physical status I, chronic intake of medication or drugs, hardness of hearing or deafness, absence of fluency in German, known or suspected disposition to malignant hyperthermia, acute hepatic porphyria, history of halothane hepatitis, obesity with a body mass index more than 30 kg/m2, gastrointestinal disorders with a disposition for gastroesophageal regurgitation, known or suspected difficult airway, and presence of metal implants.<br><br>propofol dataset: we recruited participants with posters around campus as per ethics protocol. Participants approaches the research team to seek participation, and there were no specific selection biases. Participants were required to be healthy, right-handed, native English speakers with no history of neurological disorders, and no contraindications to MRI scanning.<br><br>HCP dataset: Detailed information about the recruitment, acquisition and imaging is provided in the dedicated HCP publications.
Ethics oversight	All HCP scanning protocols were approved by the local Institutional Review Board at Washington University in St. Louis.<br>Sevoflurane dataset: The ethics committee of the medical school of the Technische Universitat Munchen (Munchen, Germany) approved the current study.<br>propofol dataset: The study received ethical approval from the Health Sciences Research Ethics Board and Psychology Research Ethics Board of Western University (Ontario, Canada).

Note that full information on the approval of the study protocol must also be provided in the manuscript.

Field-specific reporting

Please select the one below that is the best fit for your research. If you are not sure, read the appropriate sections before making your selection.

☒ Life sciences ☐ Behavioural & social sciences ☐ Ecological, evolutionary & environmental sciences

For a reference copy of the document with all sections, see nature.com/documents/nr-reporting-summary-flat.pdf

Life sciences study design

All studies must disclose on these points even when the disclosure is negative.

Sample size	This study used previously collected data. No power analysis was performed prior to data collection; the sample sizes are within the range reported in the literature.<br>sevoflurane dataset: n=20 participants were recruited .<br>propofol dataset: n=19 participants were recruited .
Data exclusions	Sevoflurane dataset: A total of 16 volunteers completed the full protocol and were included in our analyses; one participant was excluded due to high motion, leaving N=15 for analysis.<br>Propofol dataset: Due to equipment malfunction<br>or physiological impediments to anaesthesia in the scanner,<br>data from n=3 participants (1 male) were excluded<br>from analyses, leaving a total n=16 for analysis.
Replication	The propofol dataset was used to replicate all results obtained from the sevoflurane dataset. We also replicated the NeuroSynth results using the BrainMap meta-analytic dataset, and we replicated our results using a different brain parcellation (Desikan-Killiany).
Randomization	all participants were run in both conditions (awake and anaesthetised) since this was a repeated measures design.
Blinding	No blinding is possible, since anaesthetised state is tested by means of behavioural responsiveness.

Reporting for specific materials, systems and methods

We require information from authors about some types of materials, experimental systems and methods used in many studies. Here, indicate whether each material, system or method listed is relevant to your study. If you are not sure if a list item applies to your research, read the appropriate section before selecting a response.

Materials & experimental systems

n/a	Involved in the study	
☒	☐	Antibodies
☒	☐	Eukaryotic cell lines
☒	☐	Palaeontology and archaeology
☐	☒	Animals and other organisms
☒	☐	Clinical data
☒	☐	Dual use research of concern
☒	☐	Plants

Methods

n/a	Involved in the study	
☒	☐	ChIP-seq
☒	☐	Flow cytometry
☐	☒	MRI-based neuroimaging

Animals and other research organisms

Policy information about studies involving animals; ARRIVE guidelines recommended for reporting animal research, and Sex and Gender in Research

Laboratory animals	Functional MRI data were obtained from 10 exemplars of Macaca Mulatta, out of 14 (12 male, 2 female); Age distribution: 3.9-13.14 years; Weight distribution: 7.2-18 kg (full sample description available online: http://fcon_1000.projects.nitrc.org/indi/PRIME/files/newcastle.csv and http://fcon_1000.projects.nitrc.org/indi/PRIME/newcastle.html).<br><br>TheVirtualBrain project provides a dataset of preprocessed macaque fMRI comprising N=9 adult male rhesus macaques (8 Macaca mulatta, 1 Macaca fascicularis, aged between 4 and 8 years) acquired under light isoflurane anaesthesia.
Wild animals	The study did not involve wild animals.
Reporting on sex	Newcastle: Animals of both sexes were included.<br>TheVirtualBrain: male animals were used.

Field-collected samples	The study did not involve samples collected from the field.
Ethics oversight	Newcastle dataset: All of the animal procedures performed were approved by the UK Home Office and comply with the Animal Scientific Procedures Act (1986) on the care and use of animals in research and with the European Directive on the protection of animals used in research (2010/63/EU). We support the Animal Research Reporting of In Vivo Experiments (ARRIVE) principles on reporting animal research. All persons involved in this project were Home Office certified and the work was strictly regulated by the U.K. Home Office. Local Animal Welfare Review Body (AWERB) approval was obtained. The 3Rs principles compliance and assessment was conducted by National Centre for 3Rs (NC3Rs). Animal in Sciences Committee (UK) approval was obtained as part of the Home Office Project License approval.<br><br>TheVirtualBrain dataset: All surgical and experimental procedures were approved by the Animal Use Subcommittee of the University of Western Ontario Council on Animal Care and were in accordance with the Canadian Council of Animal Care guidelines, as previously reported [42].

Note that full information on the approval of the study protocol must also be provided in the manuscript.

Plants

Seed stocks	No plants used
Novel plant genotypes	No plants used
Authentication	No plants used

Magnetic resonance imaging

Experimental design

Design type	Resting-state fMRI
Design specifications	Sevoflurane dataset: five scanning sessions: awake, 2 vol%, 3 vol% burst-suppression, and recovery.<br>propofol dataset: four scanning sessions: awake, mild sedation (not considered here), deep anaesthesia, and recovery.
Behavioral performance measures	Loss of behavioural responsiveness was used to determine depth of anaesthesia.<br>Sevoflurane dataset: loss of consciousness was judged by the loss of responsiveness (LOR) to the repeatedly spoken command "squeeze my hand" two consecutive times.<br>For the propofol dataset, failure to perform two computerised tasks (a computerised auditory target-detection task and a memory test of verbal recall) was used to evaluate the level of wakefulness in the anaesthesia condition independently of the assessors, who also evaluated participants' level of behavioural responsiveness based on the Ramsay scale.

Acquisition

Imaging type(s)	Functional and anatomical
Field strength	3T for all datasets
Sequence & imaging parameters	Sevoflurane dataset: data were collected using a gradient echo planar imaging sequence (echo time = 30 ms, repetition time (TR) = 1.838 s, flip angle = 75 deg, field of view = 220 × 220 mm2, matrix = 72 × 72, 32 slices, slice thickness = 3 mm, and 1 mm interslice gap; 700-s acquisition time, resulting in 350 functional volumes). The anatomical scan was acquired before the functional scan using a T1-weighted MPRAGE sequence with 240 × 240 × 170 voxels (1 × 1 × 1 mm voxel size) covering the whole brain.<br><br>propofol dataset: MRI scanning was performed using a 3-Tesla Siemens Tim Trio scanner (32-channel coil), and 256 functional volumes (echo-planar images, EPI) were collected from each participant, with the following parameters: slices = 33, with 25% inter-slice gap; resolution = 3mm isotropic; TR = 2000ms; TE = 30ms; flip angle = 75 degrees; matrix size = 64x64. The order of acquisition was interleaved, bottom-up. Anatomical scanning was also performed,

	acquiring a high-resolution T1- weighted volume (32-channel coil, 1mm isotropic voxel size) with a 3D MPRAGE sequence, using the following parameters: TA = 5min, TE = 4.25ms, 240x256 matrix size, 9 degrees flip angle.
Area of acquisition	Whole-brain

Diffusion MRI ☒ Used ☐ Not used

Parameters: The dMRI data were from the HCP dataset. The spatial resolution was 1.25 mm isotropic. TR=5500ms, TE=89.50ms. The b-values were 1000, 2000, and 3000 s/mm2. The total number of diffusion sampling directions was 90, 90, and 90 for each of the shells in addition to 6 b0 images.

Preprocessing

Preprocessing software	Preprocessing of the functional MRI data for both datasets followed the same standard workflow as in our previous studies, and was implemented in the CONN toolbox (http://www.nitrc.org/projects/conn), version 17f [81].
Normalization	Direct normalisation to MNI space (nonlinear) using the segmented grey matter image from each volunteer's high-resolution T1-weighted image, together with an a priori grey matter template.
Normalization template	MNI-152 volumetric template, 2x2x2mm isotropic resolution.
Noise and artifact removal	Denoising followed the anatomical CompCor (aCompCor) method of removing cardiac and motion artifacts, by regressing out of each individual's functional data the first 5 principal components corresponding to white matter signal, and the first 5 components corresponding to cerebrospinal fluid signal, as well as six subject-specific realignment parameters (three translations and three rotations) and their first- order temporal derivatives, and nuisance regressors identified by the software ART 82. The subject-specific denoised BOLD signal time-series were linearly detrended and band-pass filtered between 0.008 and 0.09 Hz to eliminate both low-frequency drift effects and high-frequency noise.
Volume censoring	the artifact rejection tool (ART), implemented in the CONN toolbox, was used to identify and regress out outlying volumes, as part of the CompCor denoising procedure described above. The default CONN settings of 5 global signal z-values and 0.9mm were used.

Statistical modeling & inference

Model type and settings	We used correlation against an autocorrelation-preserving null distribution to test the spatial association between regional identifiability and canonical maps of interest. We used non-parametric permutation t-tests (repeated-measures), with 10,000 permutations to compare identifiability, cognitive matching , and similarity to the macaque regional connectivity between levels of anaesthesia,
Effect(s) tested	We tested whether anaesthesia influences identifiability; we tested whether regional identifiability is spatially associated with canonical maps of interest; we tested whether the anaesthetised FC is more similar to the macaque FC than awake FC; and we tested whether meta-analytic matching is affected by anaesthesia.

Specify type of analysis: ☒ Whole brain ☐ ROI-based ☐ Both

Statistic type for inference (See Eklund et al. 2016)	t-tests: permutation-based with 10,000 permutations. Correlation: Spearman's rank-based correlation coefficient, with p-value assessed against a null distribution of spatial autocorrelation-preserving maps.
Correction	Anaesthesia conditions were compared separately against wakefulness and against recovery, and the false positive rate against multiple comparisons was controlled using the false discovery rate (FDR) correction [10], separately for these two cases.

Models & analysis

n/a	Involved in the study
☐	☒ Functional and/or effective connectivity
☒	☐ Graph analysis
☐	☒ Multivariate modeling or predictive analysis

Functional and/or effective connectivity	Functional connectivity was obtained as the Pearson correlation between timeseries.
Multivariate modeling and predictive analysis	To consider all regional correlates together and evaluate their respective contributions, we performed a dominance analysis with all four canonical brain maps as predictors, and the regional map of anaesthetic-induced ICC changes as target.
